# Multiparameter Microwave Characterization and Probing of Ultralow Glucose Concentration Using a Microfabricated Biochip

**DOI:** 10.3390/mi7060093

**Published:** 2016-05-24

**Authors:** Kishor Kumar Adhikari, Eun Seong Kim, Nam Young Kim

**Affiliations:** RFIC Center, Kwangwoon University, 447-1 Wolgye-dong, Nowon-ku, Seoul 139-701, Korea; kishordhkr@live.com (K.K.A.); esk@kw.ac.kr (E.S.K.)

**Keywords:** complex permittivity, glucose probing, group delay, impedance at self-resonance, microfabricated biochip, multiparameter microwave characterization

## Abstract

This paper presents a planar biochip consisting of electromagnetically coupled, symmetric, square open loops for the multiparameter microwave characterization of deionized water, a phosphate-buffered saline solution, and a fructose-deionized water solution. The characterization additionally includes the probing of an ultralow glucose concentration in a very small volume of human sera and in solutions of d-glucose powder and deionized water. The interaction between the coupled electromagnetic field and the aqueous solution sample translates into a predictable relationship between the electrical characteristics of the biochip (magnitude and phase of S-parameters, attenuation, phase constant, group delay, characteristic impedance, and effective complex permittivity) and the physical properties of the solution. Owing to the microfabrication technology used for fabricating the proposed microbiochip, it is possible to develop robust, compact square open loops with a microsized coupling gap that characterizes a very small volume (1 μL) of the sample. Additionally, the biochip’s impedance peaks at its resonances were modeled using glucose-level-dependent coupling capacitance between folded square open loops and mutual inductance between center-loaded T-shaped stubs. These peaks linearly shifted in frequencies and markedly varied in impedance. Consequently, a physiologically relevant amount of glucose (50–400 mg/dL) with a high sensitivity (up to 2.036 Ω/(mg·dL^−1^)) and an ultralow detection limit (up to 4.8 nmol/L) was linearly detected.

## 1. Introduction

The spectral characterization of biomolecular aqueous solutions based on an investigation of the interaction between microwave electromagnetic (EM) fields and solution samples, as well as frequency-dependent complex-permittivity characterization of aqueous solution, serves a wide range of biomedical applications, such as the early stage detection of cancer cell [1], DNA sensing [2], cell characterization [3,4], and glucose quantification [5]. Rapid and simplified post-measurement calculations for wide-frequency spectrum-permittivity characterization with high accuracy, as well as the label-free, high-sensitivity recognition of low biomolecule concentrations in aqueous solutions using a highly miniaturized biosensor, constitute strong challenges in rapidly advancing biomedical technology.

Recently, the microwave biosensor has appeared as a promising candidate to meet these challenges. On account of the microfabrication technology used to develop high-performance microwave biosensing chips, this technology offers well-controlled critical dimensions and fluidic volumes (as low as microliters) at a relatively low cost and at high fabrication volumes without the manual-handling drawbacks. In addition, the microfabricated microwave biosensor shows great potential in offering several advantages, including label-free rapid sensing with high sensitivity, reusability, and portability. Moreover, characterization and sensing are based on the variations in S-parameters and S-parameter-based derived parameters, which appear as a response to the interaction between the biosensor’s concentrated EM field and the biomolecules under testing. Thus, these biosensors provide multidimensional sensing with improved accuracy as well as rapid wide-frequency spectrum complex-permittivity characterization [6,7].

Recently, impedance spectroscopy-based biosensors have garnered remarkable interest because of several potential advantages, such as low cost, portability, ease of application, non-invasiveness, and the capability for online monitoring. Conventional biosensors based on electrochemical impedance spectroscopy (EIS), as described by Lorenz and Schulze in 1975 [8], measure the resistive and capacitive properties of materials upon the perturbation of an electrode system by a small amplitude sinusoidal AC excitation signal over an approximate frequency range of 0.1–10 MHz. However, Fuchs and Kaatze [9] demonstrated that low-frequency (MHz) EM waves pass through the outside of a biological cell or tissue and do not affect the dielectric spectrum. Therefore, the previously reported EIS-based glucose biosensors primarily consider the less pronounced changes in resistance and capacitive reactance formed by biomolecule adsorption. Furthermore, they completely excluded changes in inductive reactance due to variations in the biomolecule level [10]. In effect, conventional EIS-based biosensors are afflicted with several limitations, including lower sensitivity, poor resolution, and a limited ability to detect lower biomolecule concentrations [11].

In this work, we therefore propose a microbiochip based on symmetrically configured, electromagnetically coupled, stub-loaded, square open loops for the label-free, rapid, highly sensitive and reproducible probing of ultralow glucose concentration, as well as the multiparameter microwave characterization of deionized water, a phosphate buffer saline (PBS) solution, and a fructose-deionized water solution. S-parameter-based analytical equations, which were used to calculate effective-complex permittivity, simplified the wide-spectral permittivity characterization. Moreover, several sensing parameters, including insertion loss (S_21_), return loss (S_11_), phase, attenuation constant, phase constant, and group delay, were simply and rapidly evaluated from the measured S-parameters to perform multiparameter sample sensing and characterization. Additionally, the characteristic impedance and its constituent parameters, including resistance, inductive reactance, and capacitive reactance, were calculated and analyzed to study the biochip’s sensing performance and determine the most sensitively varying constituent parameter at a higher frequency (GHz) regime.

The experimental results indicate that the proposed biochip exhibits glucose-level-dependent characteristic impedance with linearly shifting frequencies at its resonance peaks. The variations in impedance at resonance peaks enable the highly sensitive and reproducible detection of glucose with a nanomolar detection limit. In addition, the sensitivity of the glucose-concentration-dependent variation in inductive reactance is the highest among the impedance constituent parameters.

## 2. Materials and Methods

### 2.1. Biochip Structure, Fabrication, and Sensing Region Modeling

**Figure 1:**
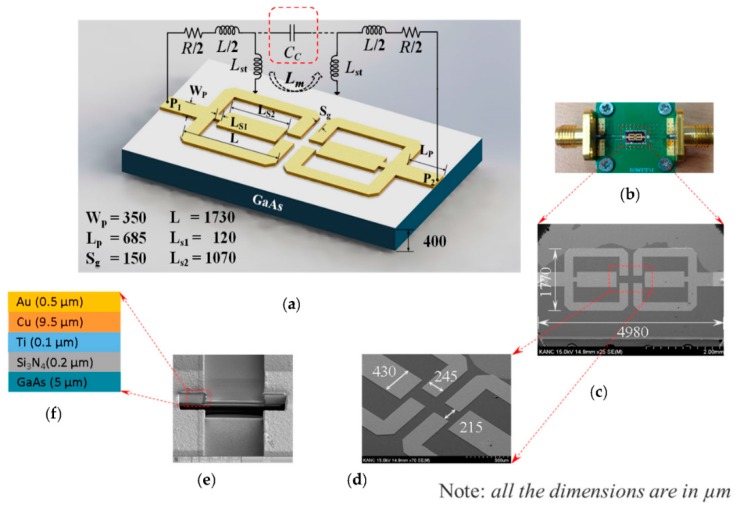
Proposed microbiochip for microwave characterization of aqueous solutions and label-free glucose probing. (**a**) Layout configuration of the biochip consisting of electromagnetically coupled, T-shaped, stub-loaded, symmetric, square open loops and its equivalent circuit, including coupling capacitance (*C_c_*) and mutual inductance (*L_m_*); (**b**) Image of the fabricated biochip mounted on printed circuit board (PCB); (**c**) Scanning electron microscopy (SEM) image of the fabricated biochip with outline dimensions; (**d**) Magnified image of a portion of the fabricated biosensor with the required dimensions; (**e**) Focused Ion Beam (FIB) image of cross-section of the fabricated biochip; (**f**) Layer information of the fabricated biochip.

The biochip layout consists of two symmetric, electromagnetically coupled, and folded square open loops, as shown in Figure 1a. The full-wave EM simulator, Sonnet, was used to draw the layout and simulate the biochip’s S-parameters. Each loop was centrally loaded with an internal T-shaped stub. The biochip’s S-parameters depended on the capacitive coupling between the open end of the proximately positioned loops and the inductive coupling between the open ends of the stubs. Therefore, the sample to be tested was located at the coupling region. The symmetrically configured square open loops were also connected to input and output ports via 50-Ω impedance-matching transmission lines.

Figure 1a additionally illustrates the equivalent circuit of the proposed biochip in terms of the net series resistive loss (*R*) of the stub-loaded square open loops with net inductance (*L*) of the loops and inductance (*L_st_*) of the T-shaped stub-loads. *C_c_* and *L_m_* represent the net coupling capacitance between the coupled portion of the loops and the net mutual inductance between the T-shaped stubs, respectively. The proposed biochip was microfabricated on a conventional 6-in 400-μm-thick gallium arsenide (GaAs) wafer with a permittivity of 12.85 and a loss tangent of 0.006 using standard microfabrication techniques. The detailed fabrication process flow of the biochip is described elsewhere [12,13].

In summary, the chip fabrication begins with the deposition of 3000-Å-thick silicon nitride (Si_3_N_4_) over the GaAs substrate as a passivation layer using plasma-enhanced chemical vapor deposition (PECVD). A 700/300-Å-thick Au/Ti seed metal layer is then sputtered to enhance the substrate adhesion to the subsequent plated metal. A photoresist is then used to mask the wafer to define the expected top metal layer structures. The defined pattern is then implemented using electroplated Cu/Au (9.5/0.5 μm). The electroplated Cu layer, used to increase the conductivity at a lower cost, may readily form an oxide layer and degrade the performance of the biochip. Therefore, it is covered with a biocompatible electroplated Au layer, which is also naturally reluctant to oxide layer formation. Figure 1b depicts the fabricated microsized chip. The chip was wire-bonded with a 50-Ω transmission line and fixed on an aluminum box. Figure 1c,d show the scanning electron microscopy (SEM) image of the fabricated biochip with its overall physical dimensions (4998 × 1770 μm^2^) and the magnified image of a portion of the chip, respectively. The cross-section image of the microfabricated biochip and its detailed layer information are shown in Figure 1e,f, respectively.

To conduct the microwave characterization experiments and performance study of the proposed biochip, the following samples were collected and prepared: deionized water (Merck Millipore, Billerica, MA, USA), a phosphate buffer saline (PBS) solution (Sigma Aldrich, Saint Louis, MO, USA), solutions of d-glucose powder (Sigma, Life Science, GC, Saint Louis, MO, USA), deionized water with a physiologically relevant amount of glucose [14] (five concentrations: 50, 100, 200, 300, and 400 mg/dL), a serum sample with a base glucose level of 100 mg/dL, samples consisting of various glucose levels obtained by the addition of solutions of d-glucose powder and deionized water (5 concentrations: 100, 125, 150, 175, and 200 mg/dL), and a sample consisting of solutions of d-fructose powder and deionized water (20 mg/dL). An Agilent 8510C vector network analyzer (VNA) was used to measure the biochip’s S-parameters. It was calibrated using the short, open, load, through (SOLT) technique to ensure the accuracy and repeatability of the measurements.

### 2.2. Modeling of Electromagnetically Coupled Sensing Region

**Figure 2:**
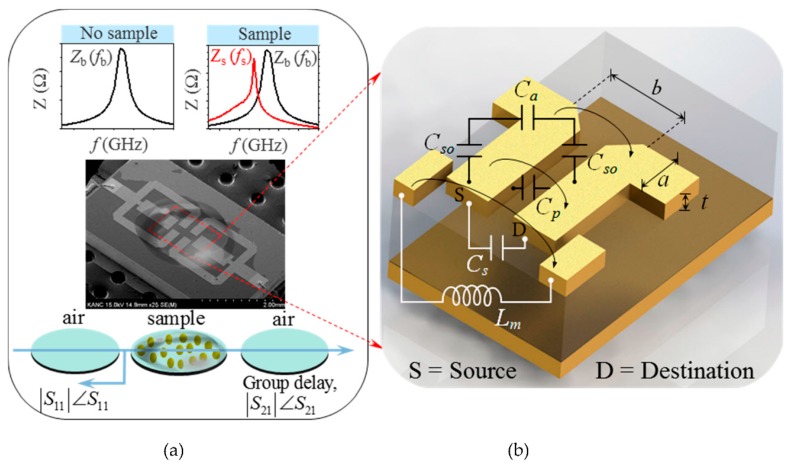
Microwave characterization principle and modeling of sensing region. (**a**) Scanning electron microscope (SEM) image of the fabricated biochip illustrating its sample characterization principle in a microwave regime based on S-parameters, derived parameters, and characteristic impedance self-resonance spectroscopy; (**b**) Equivalent circuit modeling of the sensing region in terms of its sample-dependent coupling capacitance and mutual inductance.

The incident EM wave, which moves from the left, as depicted in Figure 2a, passes through the air to incise in the material during a test phase through the transition. Once in the material, the wave velocity is slower, and the wavelength is shorter. Attenuation and insertion loss occur on account of the presence of a lossy material. Because the impedance of the wave in the material is different (lower) than the free space impedance, an impedance mismatch occurs, which creates the reflected wave. Part of the energy penetrates the sample. As a result, incident, reflected, and transmitted waves occur. The characteristics of these waves depend on the equivalent coupling capacitance and mutual inductance, and are guided by the nature of the material under testing. Therefore, these waves play an important role in studying the material characteristics under a microwave regime.

According to the microwave polarization theory (detailed in our previous work [15]), the interaction of EM waves under a microwave regime with an aqueous sample tends to move the charge in the sample and reorient the dipoles. It consequently forms a quadruple. The local field corresponding to the quadruple results in the effective permittivity of the biochip. Accordingly, the net coupling capacitance and mutual inductance of the system result. Mathematically, the net coupling capacitance of the electromagnetically coupled portions of the biochip’s square open loops in the presence of the sample is expressed using the following equation:

(1) $$C_{c}=C_{s}+C_{p}+{\left[\frac{1}{C_{so}}+\frac{1}{C_{a}}\right]}^{-1}$$

where $C_{p}\left[=\frac{\varepsilon_{0}\varepsilon_{s}K\cdot \sqrt{1-K^{2}}}{K(k)}\right]$, and *C_s_* (= ε*_o_*ε*_so_t*/*a*) respectively denote the capacitive effects due to the direct flux and flux via the substrate from the source to destination loop, as shown in Figure 2b. In addition, *k* = *a*/*b* (= 0.6125) and *K*(*k*) (= 1.76) is the elliptical integral of the first kind. $C_{a}\left[=\frac{\varepsilon_{0}K\cdot \sqrt{1-K^{2}}}{K(k)}\right]$ and $C_{so}\left[=\frac{\varepsilon_{0}\varepsilon_{so}K\cdot \sqrt{1-K^{2}}}{K(k)}\right]$ respectively represent the capacitive effects due to flux in the air and the solution sample. Here, ε*_o_* represents air permittivity, ε*_s_* is the substrate permittivity, and ε*_so_* is the permittivity of the solution sample. Similarly, the net mutual inductance (*L_m_*) between the coupled T-shaped stubs of the biochip in the presence of the sample is expressed using the following equation:

(2) $$L_{m}=\frac{\mu_{so}\varepsilon_{so}}{C_{st}}$$

where μ*_so_* and ε*_so_* represent the effective permeability and permittivity of the solution sample, respectively, and *C_st_* is the capacitive effect between the coupled stubs. The symmetric structure of the proposed biochip enables analysis of its characteristic impedance (*Z_c_*), including the effects of coupling parameters, in terms of its odd-mode (*Z_oo_*) and even-mode impedance (*Z_oe_*) expressed using the following equations [16]:

(3) $$Z_{oe}=\sqrt{\frac{R+j\omega (L_{o}-L_{m})}{G+j\omega (C_{o}-2C_{c})}}$$

(4) $$Z_{oo}=\sqrt{\frac{R+j\omega (L_{o}-L_{m})}{G+j\omega (C_{o}+2C_{c})}}$$

and

(5) $$Z_{c}=\sqrt{Z_{oe}\cdot Z_{oo}}$$

## 3. Results and Discussion

### 3.1. Biochip Characterization for Aqueous Solutions

**Figure 3:**
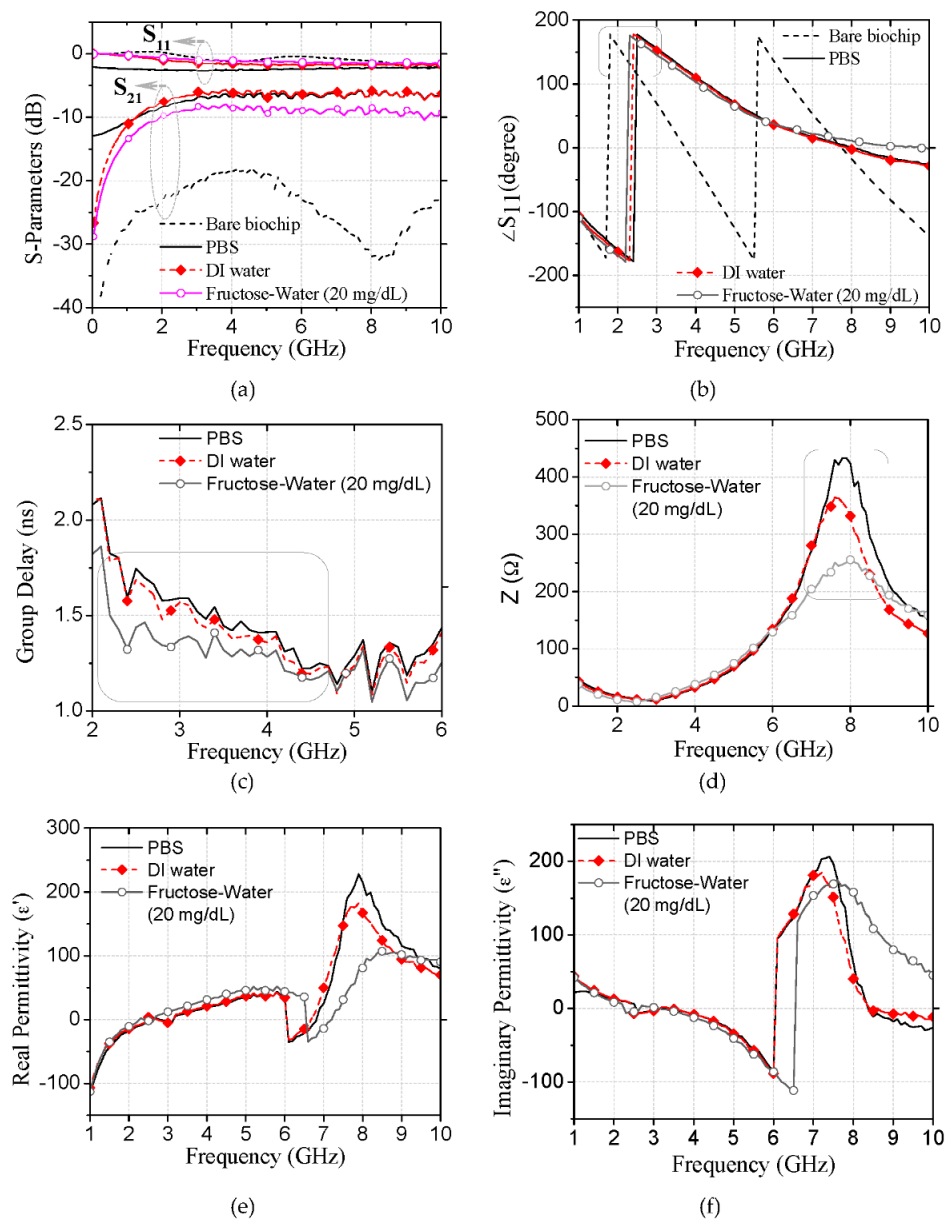
Microwave characterization of the proposed biochip for PBS, DI water, and the d-fructose-DI water solution. (**a**) S-parameters for the bare biochip and biochip bearing a 1-μL sample; (**b**) phase of S_11_; (**c**) group delay; (**d**) self-resonating impedance; (**e**) real permittivity; (**f**) imaginary permittivity.

The measured return loss (S_11_) and insertion loss (S_21_) of the bare biochip and the biochip bearing deionized water, the phosphate-buffered saline (PBS) solution, and the fructose–water solution—which are plotted against a frequency range of 0.1–10 GHz in Figure 3a—indicate a significant increase and decrease in the reflected and transmitted energy of the bare biochip relative to the biochip consisting of the sample. Additionally, maximum and minimum transmitted energies are observed for the PBS and fructose–water solution, respectively. It should be noted that the PBS solution is the most viscous material among the tested materials [17]. However, the fructose–water solution exhibits the lowest viscosity [18] among them because the addition of fructose in water lowers its viscosity. Therefore, the physical properties (viscosities) of the tested solutions exhibit a manifest and informative correlation with the reflected and transmitted energies of the biochip. The phase of S_11_, which is shown in Figure 3b for the above conditions, indicates that the frequency corresponding to the 180° phase of the biochip bearing the sample is shifted upward. This upward frequency shift is the highest and lowest for the PBS solution and fructose–water solution, respectively.

The group delay of S_21_ (= dφ/dω, where φ and ω represent the phase and angular frequency of S_21_, respectively) for the biochip bearing different solution samples (Figure 3c) indicate the most and least delayed signal for the PBS and fructose–water solution sample. Figure S1a shows that the biochip transmitted signal attenuation is maximized and minimized for the PBS and fructose–water solution, respectively. Additionally, the PBS solution exhibits the least phase constant, as displayed in Figure S1b. However, there is no significant difference between the biochip phase constant for the deionized water and aqueous fructose.

To further characterize the solution samples, the characteristic impedances of the biosensor with various samples were calculated from the measured S-parameters using the following equation [19]:

(6) $$Z_{c}=\pm Z_{o}\sqrt{\frac{{\left(1+S_{11}\right)}^{2}-S_{21}^{2}}{{\left(1-S_{11}\right)}^{2}-S_{21}^{2}}}$$

The calculated characteristic impedances, which are plotted against a frequency range of 0.1–10 GHz in Figure 3d, indicate that the biochip exhibits a resonance in impedance for each sample. The impedance and frequency at resonance peak are unique for each sample. Moreover, the impedance at peak is the highest for the PBS solution and lowest for the fructose–water solution. In summary, all of the above parameters indicate a unidirectional change for the above-mentioned solution samples.

To study the glucose-level-dependent characteristic impedance resonance phenomena, the ε*_eff_* of the glucose biosensor for glucose samples of varying concentrations were calculated using the following equation [20]:

(7) $$\varepsilon_{eff}=n/z$$

where *z* (= Z*_c_*/50) and *n* is the normalized impedance and refractive index, respectively. Furthermore, *n* was evaluated based on the measured S-parameters using the following equation [21]:

(8) $$n=\pm \frac{1}{kd}\cos^{-1}\left[\frac{1}{2S_{21}}\left(1-S_{11}^{2}+S_{21}^{2}\right)\right]$$

where *k* (= ω/*c*) and *d* (= 4.98 mm) are the wave number and propagation distance of the incident wave, respectively, and *c* is the speed of light in a vacuum.

Figure 3a illustrates the real permittivity (ε*_eff_*’) for various aqueous solution samples. As shown in the figure, ε*_eff_*’ successively transforms from negative to positive values and exhibits peaks within an approximate frequency range of 7.5–9 GHz. The frequencies of the ε*_eff_*’ peaks are the highest and lowest for the aqueous fructose and PBS solutions, respectively. However, the magnitudes of the ε*_eff_*’ peaks were maximized and minimized for the PBS and aqueous fructose solutions, respectively. The imaginary permittivity (ε*_eff_*’’), shown in Figure 3f for various samples, also exhibits resonance peaks that indicate a manifest correlation with the sample type.

### 3.2. Glucose Probing Using Characteristic Impedance at Its Resonances

**Figure 4:**
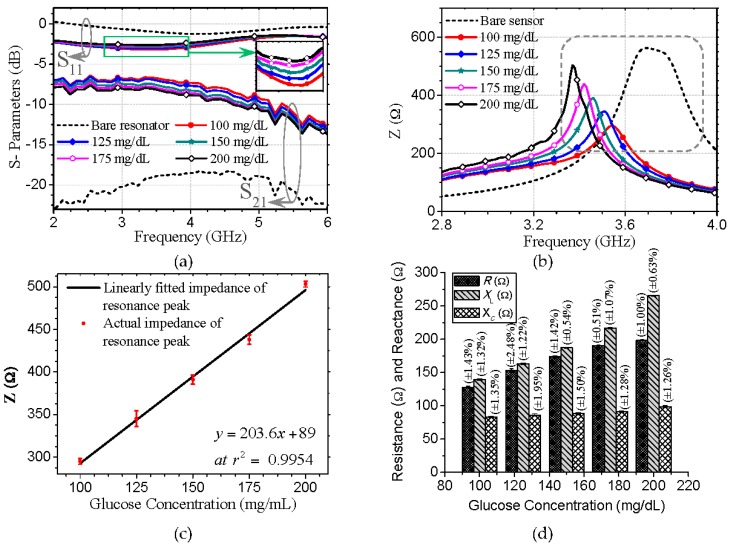
Microwave impedance spectroscopy-based label-free detection of glucose in human sera. (**a**) Glucose-level-dependent S-parameters of the proposed biochip; (**b**) The biochip’s glucose-level-dependent characteristic impedances and their resonances for human sera of varying glucose concentrations (100–200 mg/dL); (**c**) Linearly fitted impedances of the resonance peaks, including actual impedances of the peaks and error bars (*n* = 6) represented by RSD; (**d**) Bar diagram indicating actual resistance (*R*), inductive capacitance (*X_L_*), and capacitive reactance (*X_C_*) at impedance resonance frequencies for varying glucose concentrations, including error bars (*n* = 6).

The S-parameters of the proposed biochip, which were measured for serum samples of varying glucose concentrations in a frequency range of 2–6 GHz, are depicted in Figure 4a. The results indicate that the reflected and transmitted energies are respectively maximized and minimized when the concentration of glucose in the serum sample is maximized. However, this correlation is only clearly pronounced for an approximate frequency range of 2–5 GHz.

To detect the levels of glucose based on impedance spectroscopy, the characteristic impedances of the biosensor with glucose samples of varying concentrations were calculated from the measured S-parameters using Equation (6). The calculated impedances, which are plotted against frequency in Figure 4b, indicate a marked positive correlation between the impedance and glucose level for an approximate frequency range of 3–3.7 GHz. In addition, the impedances exhibit glucose level-dependent resonance peaks, whose variations in impedance are more sensitive than those of any other points. The impedance of the resonance peaks with the minimum and maximum glucose concentrations of 100 and 200 mg/dL are 295 and 503 Ω, respectively. For other glucose samples, the peak impedance is maximized when the concentration of glucose in the serum is maximized. The solid line in Figure 4c, plotted using a least square regression method, represents a linear fit (*r*^2^ = 0.9954; *r* is the coefficient of correlation) of the glucose concentration (*x*) with the variations in the impedance of peaks (*y*) using the following equation:

(9) y=2.036x+89

Therefore, the glucose probing sensitivity of the proposed biochip in human sera, as indicated by the slope of Equation (9), is high (2.036 Ω/(mg·dL^−1^)). The detection limit, estimated at a signal-to-noise ratio (S/N) of three, as previously outlined [22], was found to be 4.8 nmol glucose in a 1-μL sample. The relative standard deviations (RSDs) of sextuplicate impedance measurements for the individual sample and deviation points from the impedance peak are represented by nonoverlapping error bars. The maximum RSD of 4.37% for a glucose sample of 200 mg/dL is exhibited by the biosensor. It indicates the high reproducibility of the glucose concentration data generated by this biochip for human sera. The frequencies of the impedance self-resonance peaks shift downward for various glucose samples. The amount of shift with respect to the bare sensor impedance peak at 3.72 GHz indicates a clear dependence on the glucose level. The frequencies of the peaks at the minimum and maximum glucose concentrations of 100 and 200 mg/dL are 3.54 and 3.37 GHz, respectively. Thus, the downward shift in the frequency of the biochip’s impedance peak is maximized when the concentration of glucose in the serum is maximized.

The regression analysis reveals a good linear correlation (*r*^2^ = 0.99) between the glucose concentration (*x*) and shift in frequency (*f*) of the impedance peaks expressed as *f* = −0.00172*x* + 3.718. Therefore, the biochip exhibits a sensitivity of 1.72 MHz/(mg·dL^−1^). According to the optimization study, the detection limit of the assay for an S/N of three was calculated to be 0.24-μmol glucose in a 1-μL sample. The maximum RSD of 0.61% obtained by the biosensor for a glucose sample of 100 mg/dL out of six experiments indicates that the glucose detection mechanism yields highly reproducible data. Therefore, both the variation in impedance and shift in the frequency of resonance peaks enable the very sensitive detection of glucose in human sera. The variations in impedances increase the sensitivity, while the shift in frequency improves the reproducibility. Table 1 presents the values of the impedances and frequencies of the resonance peaks with the respective RSDs of the proposed biochip for serum samples of varying glucose concentrations. The proposed biochip exhibits a glucose detection limit at least 19 times lower than several previously reported EIS-based glucose biosensors [23,24,25,26,27,28,29,30].

The variations in the resistance of the biochip with glucose samples of varying concentrations, illustrated in Figure S2a, indicate that the resistance of the biochip crosses from a negative at lower frequencies (approximately 3–3.4 GHz) to a positive value and exhibits resonance peaks at frequencies slightly higher than the frequencies of impedance resonance peaks. The resistance at resonance peaks also positively correlates with the glucose level. This behavior is expected because the conductance of the glucose sample positively correlates with the glucose level.

The resistances of the resonance peaks are 127.5 and 197.8 Ω at the minimum and maximum glucose concentrations of 100 and 200 mg/dL, respectively, as shown by the bar diagram in Figure 4d. The regression analysis reveals a good linear correlation (*r*^2^ = 0.97) between the glucose concentration and variation in peak resistance with a sensitivity of 0.712 Ω/(mg·dL^−1^) and a reproducibility of maximum RSD of 2.48% for a serum sample with a glucose concentration of 125 mg/dL. This finding suggests that a relatively smaller portion (35.44%) of the variation in impedance at its resonance is due to the variation in resistance, while the remaining portion of the variation is due to the variation in reactance. Figure S2b and Figure 4d respectively show the inductive reactance (*X_L_* = ω*L*) for a range of frequencies and at impedance resonance peak frequencies for serum samples of varying concentrations. These figures indicate that *X_L_* exhibits peak values of 144.8 and 269.4 Ω for glucose levels of 100 and 200 mg/dL, respectively. In addition, *X_L_* peaks when its resonances positively correlate with the glucose level. Regression analysis revealed that the *X_L_* varies linearly (*r*^2^ = 0.972) with glucose concentration and exhibits both a sensitivity of 1.222 Ω/(mg·dL^−1^) and a reproducibility of maximum RSD of 1.32% for a serum sample with a glucose concentration of 100 mg/dL. Therefore, approximately 58.91% of the variation in impedance at its resonance is due to the variation in inductive reactance.

Figure 4d shows a positive correlation between the capacitive reactance (*X_C_* = 1/ω*C*) at impedance resonance peak frequencies and varying glucose levels of serum samples (peak values of 83.2 and 95.7 Ω for glucose levels of 100 and 200 mg/dL, respectively). This result indicates that the variation of *X_C_* exhibits a linear relation (*r*^2^ = 0.921) with glucose level, a sensitivity of 0.1456 Ω/(mg·dL^−1^), and a reproducibility of maximum RSD of 1.95% for a serum sample with a glucose concentration of 100 mg/dL. Therefore, approximately 5.8% of the impedance variation is due to the variation in capacitive reactance. Thus, the impedance of the proposed biosensor exhibits resonances when the sensitive variations of the resistance at its resonances, inductive reactances, and insensitive variations in capacitive reactance are combined. In summary, the resonance in characteristic impedance of the proposed biochip, whose variations are most sensitively caused by variation in inductive reactance at a microwave regime, enables a highly sensitive and reproducible probing of ultralow glucose concentration.

**Figure 5:**
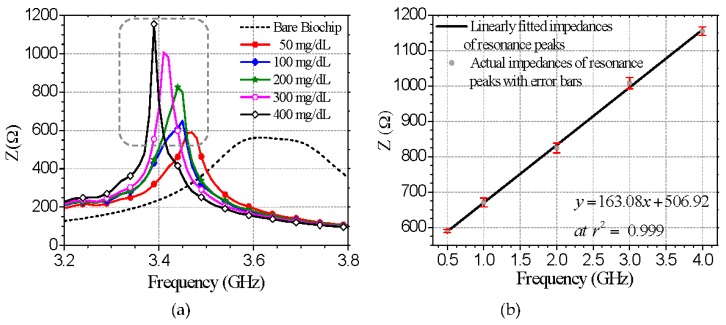
Microwave-impedance spectroscopy-based label-free detection of glucose in deionized water and d-glucose powder solutions. (**a**) Glucose-level dependent characteristic impedances of the proposed glucose biosensor for glucose-water solutions of varying glucose concentrations (50–400 mg/dL); (**b**) Linearly fitted impedances of the resonance peaks, including actual impedances of the peaks and error bars represented by RSDs of six independent measurements

The measured glucose-level-dependent impedances of the biosensor for five samples of glucose-water solutions, with the glucose level ranging from 50–400 mg/dL, are shown in Figure 5a. The nature of the variation in impedance and the shift of frequency of the impedance at its resonance peaks are similar to these values in the serum samples. The regression analysis reveals a good linear correlation (*r*^2^ = 0.999) between the glucose concentration and impedance variation at the resonance peaks with the following linear regression:

(10) y=1.6308x+506.92

Therefore, the biosensor exhibits a sensitivity of 1.6308 Ω/(mg·dL^−1^) for water–glucose solutions. According to the results of the optimization study and associated calibration plot with error bars (see Figure 5b), the detection limit of the assay for S/N = 3 was calculated as 0.0345 μmol of glucose in 1 μL of sample. Moreover, the maximum RSD of 1.609% at the glucose level of 300 mg/dL, indicated by the following figures.

### 3.3. Biochip Detection Accuracy against Interference of Fructose

To evaluate the practicality of the present glucose-sensing method, the biochip was used to detect the levels of glucose in human serum samples from three human subjects. The impedance of the biosensor for 1 μL of each sample was calculated, and the concentration of the glucose in the serum was determined based on the impedance peak value using the calibration of Equation (9). The results are shown in Table 2. It is clear that the glucose concentrations determined using the proposed method are consistent with those determined using the clinically established method. The RSD is lower than 3.28%, and the average recoveries of glucose in the real samples ranged from 98.91% to 101.26%. These results indicate that the proposed biosensor accurately measures the levels of glucose and can therefore be applied for a real sample assay.

To test the selectivity of the proposed glucose biochip against the potential interfering glucose analogs, each serum sample was then supplemented with 20 mg/dL of fructose. Fructose was selected as an interfering agent on account of its larger dynamic ranges depending on the patient’s diet. The recovered levels of glucose in serum samples with fructose using the proposed biochip are displayed in Table 1. The results indicate a less than 1.26% change with a maximum RSD of 3.79%; therefore, the selectivity of the biochip is satisfactory.

## 4. Conclusions

In this work, we developed a microfabricated biochip based on electromagnetically coupled, symmetric, stub-loaded, square open loops for the multiparameter microwave characterization of deionized water, a PBS solution, a solution of fructose and deionized water, a water–glucose solution, and human sera. Unidirectional changes in magnitude and the phase of S-parameters, attenuation, phase constant, group delay, characteristic impedance resonance peak, and changes in complex permittivity successfully characterize the sample type and indicated their usefulness in identifying relatively viscous material. Moreover, the biochip’s characteristic impedance at its resonances linearly and accurately detect the level of glucose in human sera against the interference of fructose as well as a physiologically relevant amount of glucose in water–glucose solutions with high sensitivity and an ultralow glucose detection limit (up to 4.98 nmol in a 1-μL glucose sample). Therefore, the satisfactory performance of the proposed microbiochip constitutes a new, qualified alternative to complex permittivity characterization and detection of glucose based on spectroscopy of characteristic impedance at its resonance in practical and routine analyses. Additionally, it was determined that the variation in the biochip’s microwave characteristic impedance at resonance peaks—including unidirectional sensitive variations in the resistance at resonances and inductive reactances, as well as changes in the capacitive reactance—indicate that the most sensitive variation in inductive reactance is due to the variation in glucose concentration.

## Figures and Tables

**Table 1:** Variation in magnitude and shift of frequency of the biochip’s characteristic impedance at its resonance for serum samples of varying glucose concentrations.

Concentration * (mg·dL^−1^)	Magnitude of Impedance Peak (Ω)	± RSD ^#^ (%)	Frequency of Impedance Peak (GHz)	± RSD (%)
Bare biochip	562	0.00	3.72	0.00
100	295	3.66	3.54	0.61
125	345	4.21	3.51	0.60
150	391	4.35	3.46	0.14
175	438	1.66	3.42	0.50
200	503	4.37	3.37	0.16

* Each concentration sample was measured six times. ^#^ RSD = Relative standard deviation.

**Table 2:** Determination of glucose level in serum samples (*n* = 3) for specificity analysis.

Sample *	Determined by Clinically Established Method (mg/dL)	Determined by Biosensor (mg/dL) ± RSD (%)	Supplemented Fructose (mg/dL)	± RSD (%)	Recovery (%)
1	190	191.51 ± 1.89	0.2	191.51 ± 2.40	100.79
2	134	135.69 ± 3.28	0.2	135.69 ± 3.79	101.26
3	156	154.30 ± 2.54	0.2	154.30 ± 3.09	98.91

* Each sample was measured six times.

## Supplementary Materials

The following are available online at http://www.mdpi.com/2072-666X/7/6/93/s1. Figure S1: Microwave characterization of aqueous solutions using proposed microfabricated biochip. (a) Attenuation and (b) phase constant. Figure S2: Glucose-level-dependent impedance constituent parameters of the proposed biochip for human sera. (a) Net resistances and (b) net inductive reactance.
