# Supplementary material for: Multiparameter Microwave Characterization and Probing of Ultralow Glucose Concentration Using a Microfabricated Biochip
Source: Micromachines (Basel). 2016 May 24;7(6):93. doi: 10.3390/mi7060093 (PMC6189731; doi:10.3390/mi7060093)
Supplement: Supplementary file 1 [file micromachines-07-00093-s001.pdf]

Supplementary Materials: Multiparameter Microwave Characterization and Probing of Ultralow Glucose Concentration Using Microfabricated Biochip

Kishor Kumar Adhikari, Eun Seong Kim and Nam Young Kim *

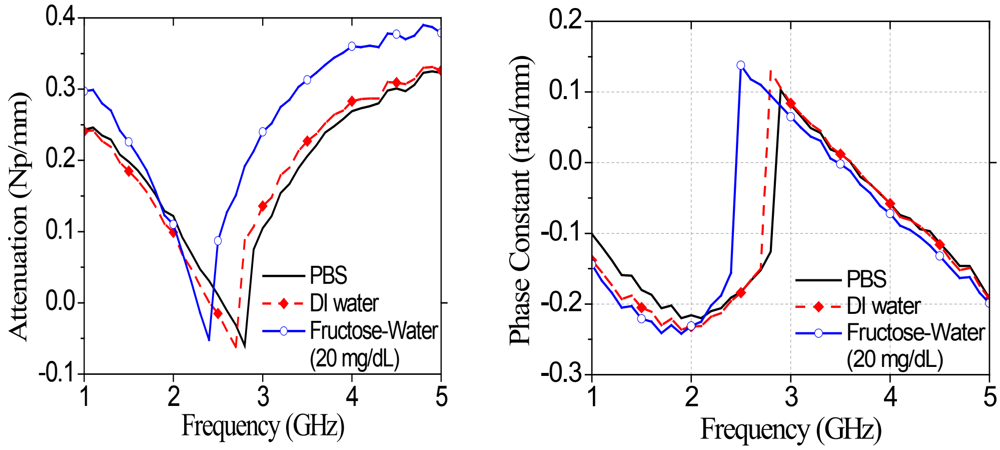

(**a**) (**b**)

Figure S1. Microwave characterization of aqueous solutions using proposed microfabricated biochip. (**a**) Attenuation and (**b**) phase constant.

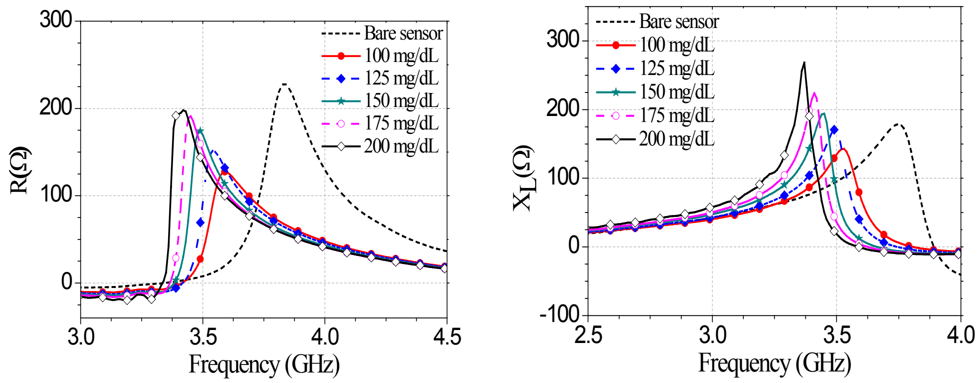

(**a**) (**b**)

Figure S2. Glucose-level-dependent impedance constituent parameters of the proposed biochip for human serum. (**a**) Net resistance and (**b**) net inductive reactance.
